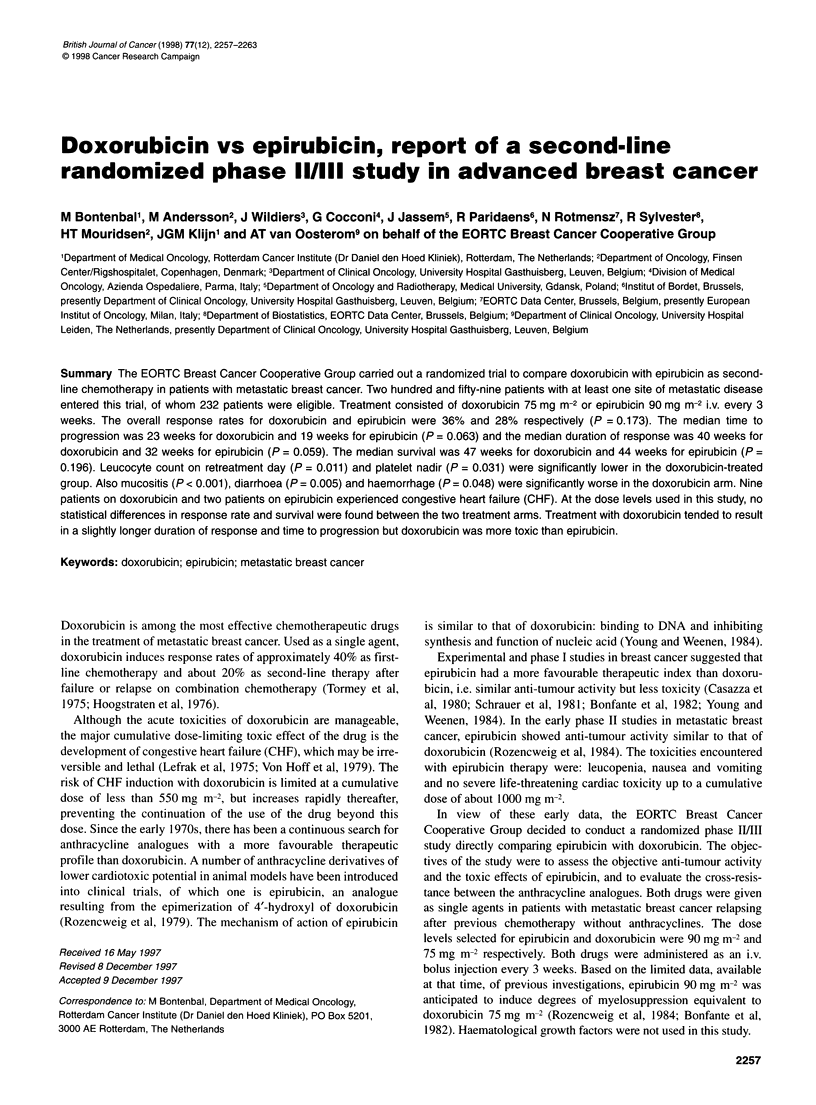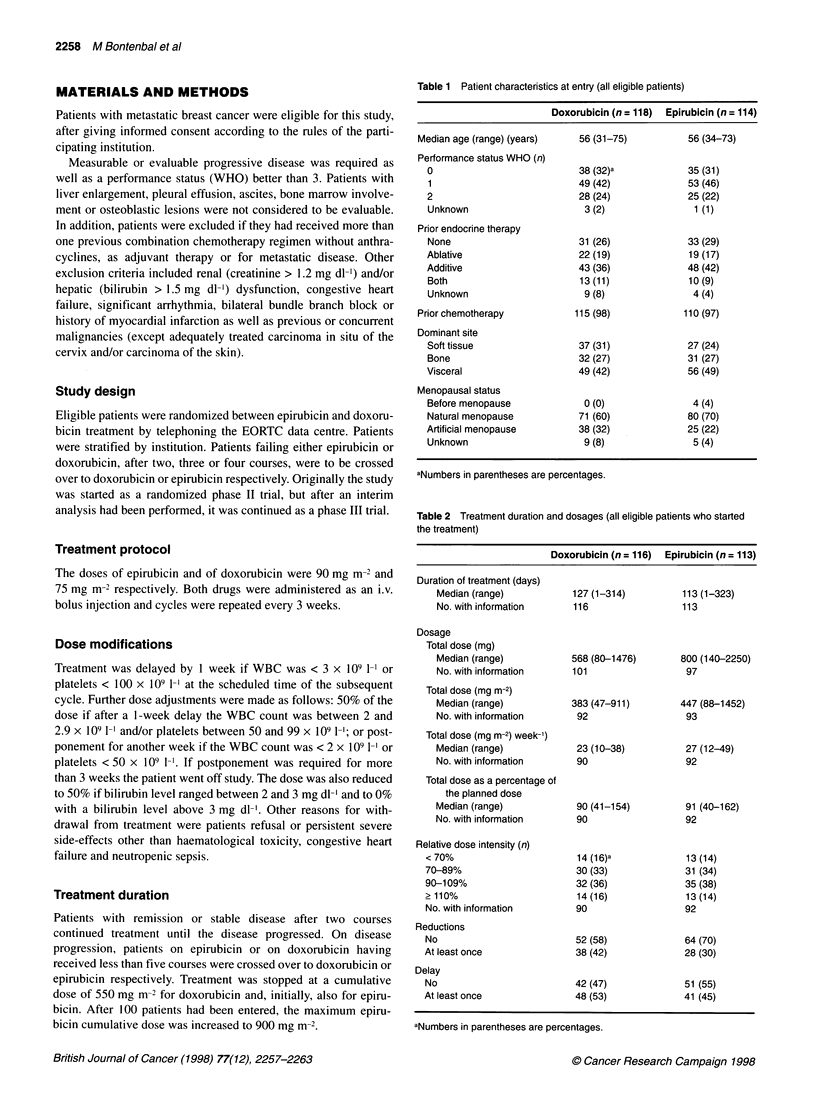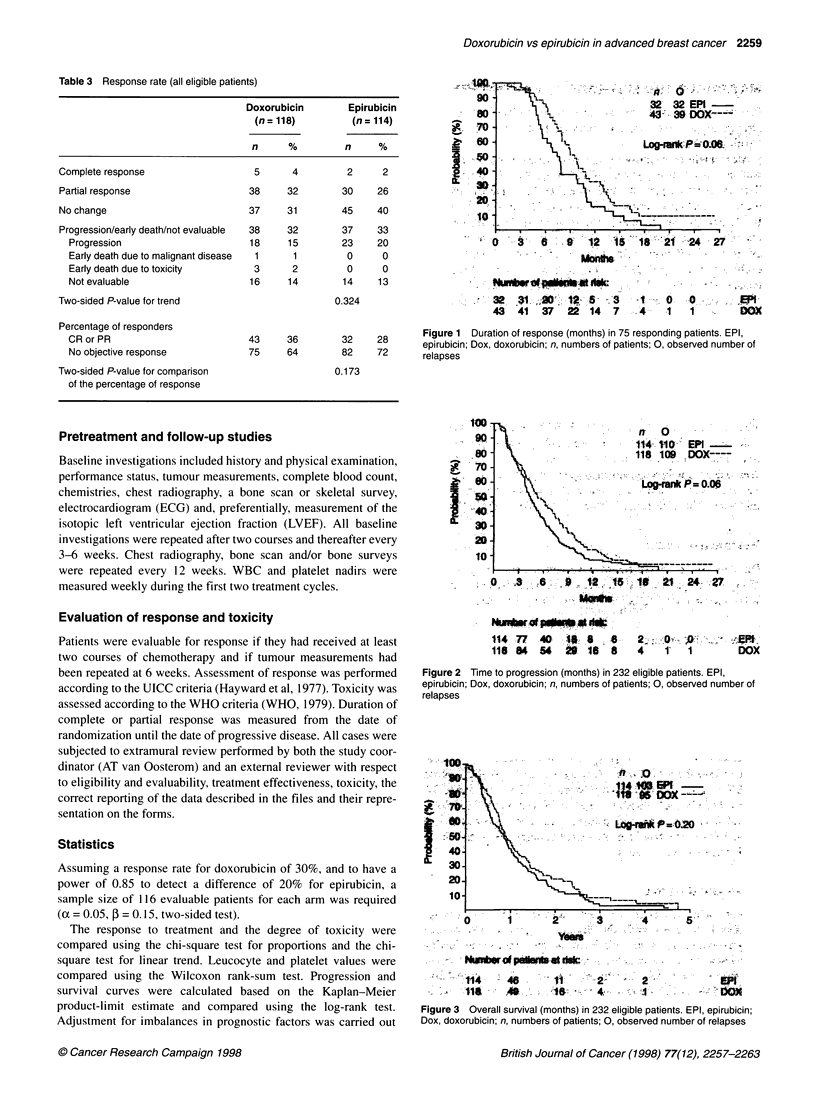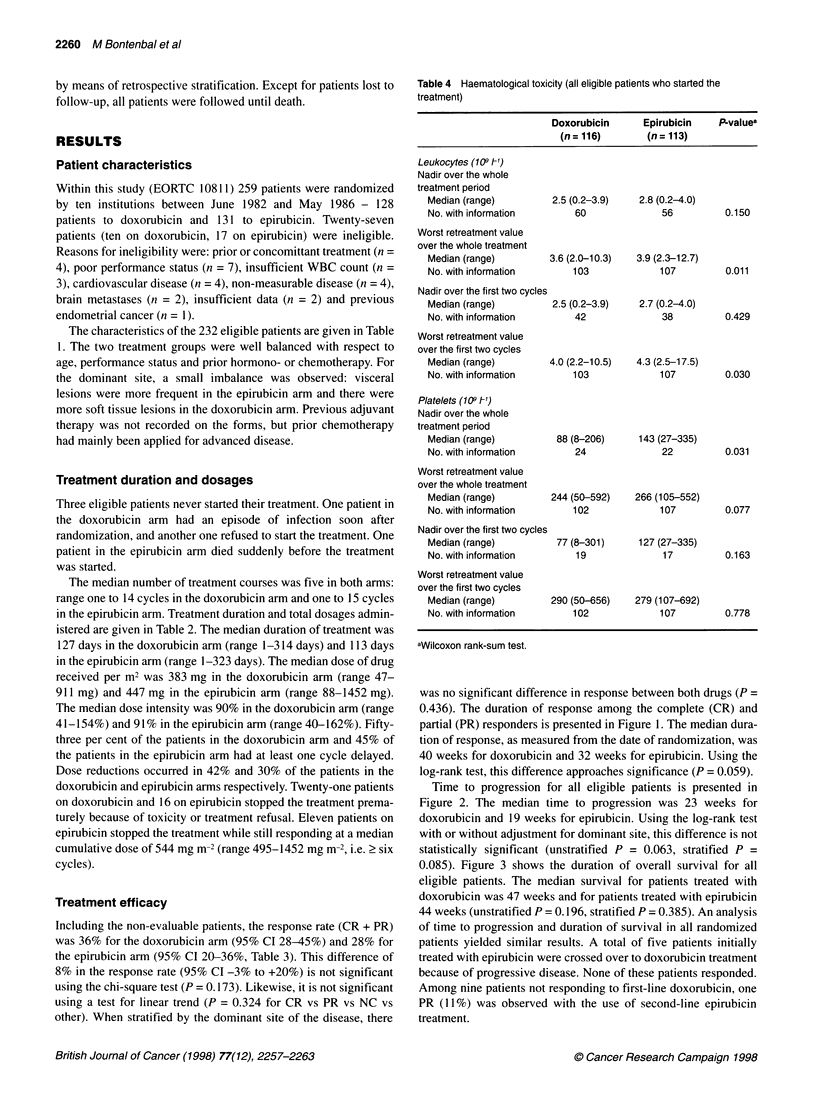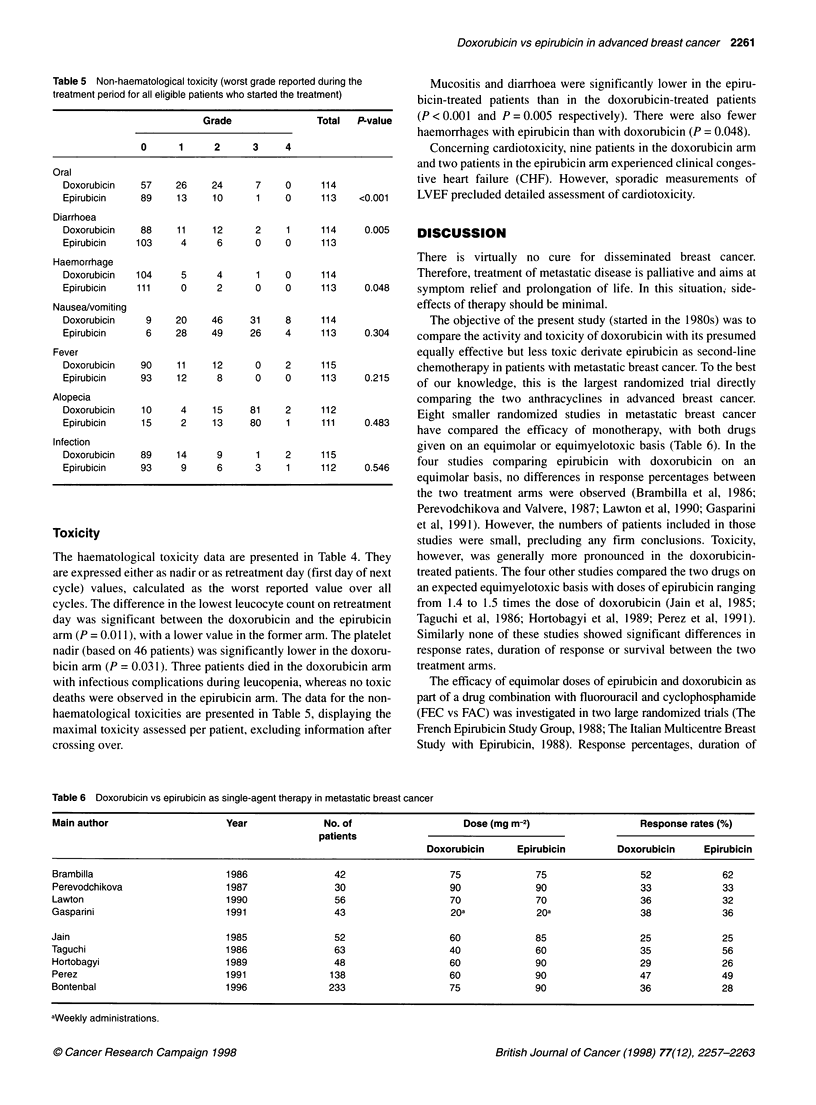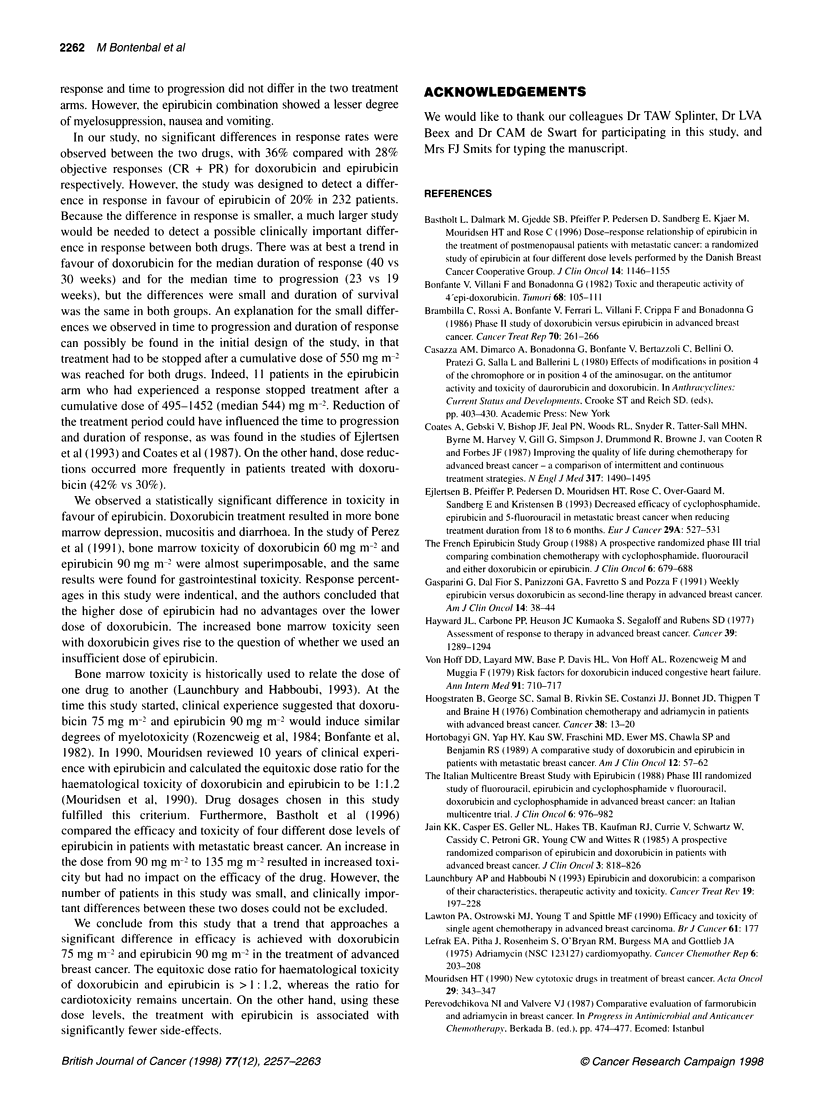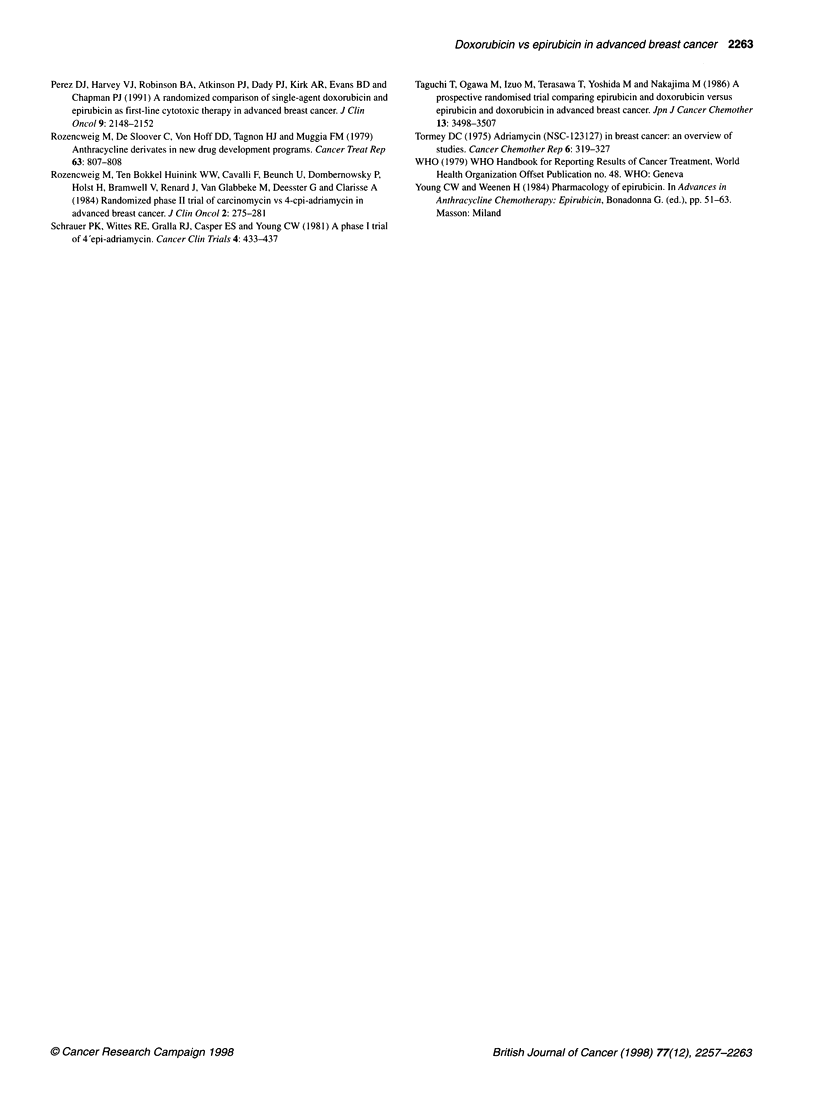# Doxorubicin vs epirubicin, report of a second-line randomized phase II/III study in advanced breast cancer. EORTC Breast Cancer Cooperative Group.

**DOI:** 10.1038/bjc.1998.375

**Published:** 1998-06

**Authors:** M. Bontenbal, M. Andersson, J. Wildiers, G. Cocconi, J. Jassem, R. Paridaens, N. Rotmensz, R. Sylvester, H. T. Mouridsen, J. G. Klijn, A. T. van Oosterom

**Affiliations:** Department of Medical Oncology, Rotterdam Cancer Institute (Dr Daniel den Hoed Kliniek), The Netherlands.

## Abstract

The EORTC Breast Cancer Cooperative Group carried out a randomized trial to compare doxorubicin with epirubicin as second-line chemotherapy in patients with metastatic breast cancer. Two hundred and fifty-nine patients with at least one site of metastatic disease entered this trial, of whom 232 patients were eligible. Treatment consisted of doxorubicin 75 mg m(-2) or epirubicin 90 mg m(-2) i.v. every 3 weeks. The overall response rates for doxorubicin and epirubicin were 36% and 28% respectively (P = 0.173). The median time to progression was 23 weeks for doxorubicin and 19 weeks for epirubicin (P = 0.063) and the median duration of response was 40 weeks for doxorubicin and 32 weeks for epirubicin (P = 0.059). The median survival was 47 weeks for doxorubicin and 44 weeks for epirubicin (P = 0.196). Leucocyte count on retreatment day (P = 0.011) and platelet nadir (P = 0.031) were significantly lower in the doxorubicin-treated group. Also mucositis (P < 0.001), diarrhoea (P = 0.005) and haemorrhage (P = 0.048) were significantly worse in the doxorubicin arm. Nine patients on doxorubicin and two patients on epirubicin experienced congestive heart failure (CHF). At the dose levels used in this study, no statistical differences in response rate and survival were found between the two treatment arms. Treatment with doxorubicin tended to result in a slightly longer duration of response and time to progression but doxorubicin was more toxic than epirubicin.


British Journal of Cancer (1998) 77(12), 2257–2263
© 1998 Cancer Research Campaign

### Doxorubicin vs epirubicin, report of a second-line randomized phase II/III study in advanced breast cancer

**M Bontenbal[1], M Andersson[2], J Wildiers[3], G Cocconi[4], J Jassem[5], R Paridaens[6], N Rotmensz[7], R Sylvester[8], HT Mouridsen[2], JGM Klijn[1] and AT van Oosterom[9] on behalf of the EORTC Breast Cancer Cooperative Group**

[1]Department of Medical Oncology, Rotterdam Cancer Institute (Dr Daniel den Hoed Kliniek), Rotterdam, The Netherlands; [2]Department of Oncology, Finsen Center/Rigshospitalet, Copenhagen, Denmark; [3]Department of Clinical Oncology, University Hospital Gasthuisberg, Leuven, Belgium; [4]Division of Medical Oncology, Azienda Ospedaliere, Parma, Italy; [5]Department of Oncology and Radiotherapy, Medical University, Gdansk, Poland; [6]Institut of Bordet, Brussels, presently Department of Clinical Oncology, University Hospital Gasthuisberg, Leuven, Belgium; [7]EORTC Data Center, Brussels, Belgium, presently European Institut of Oncology, Milan, Italy; [8]Department of Biostatistics, EORTC Data Center, Brussels, Belgium; [9]Department of Clinical Oncology, University Hospital Leiden, The Netherlands, presently Department of Clinical Oncology, University Hospital Gasthuisberg, Leuven, Belgium

**Summary** The EORTC Breast Cancer Cooperative Group carried out a randomized trial to compare doxorubicin with epirubicin as second-line chemotherapy in patients with metastatic breast cancer. Two hundred and fifty-nine patients with at least one site of metastatic disease entered this trial, of whom 232 patients were eligible. Treatment consisted of doxorubicin 75 mg $m^{-2}$ or epirubicin 90 mg $m^{-2}$ i.v. every 3 weeks. The overall response rates for doxorubicin and epirubicin were 36% and 28% respectively ($P$ = 0.173). The median time to progression was 23 weeks for doxorubicin and 19 weeks for epirubicin ($P$ = 0.063) and the median duration of response was 40 weeks for doxorubicin and 32 weeks for epirubicin ($P$ = 0.059). The median survival was 47 weeks for doxorubicin and 44 weeks for epirubicin ($P$ = 0.196). Leucocyte count on retreatment day ($P$ = 0.011) and platelet nadir ($P$ = 0.031) were significantly lower in the doxorubicin-treated group. Also mucositis ($P$ < 0.001), diarrhoea ($P$ = 0.005) and haemorrhage ($P$ = 0.048) were significantly worse in the doxorubicin arm. Nine patients on doxorubicin and two patients on epirubicin experienced congestive heart failure (CHF). At the dose levels used in this study, no statistical differences in response rate and survival were found between the two treatment arms. Treatment with doxorubicin tended to result in a slightly longer duration of response and time to progression but doxorubicin was more toxic than epirubicin.

**Keywords:** doxorubicin; epirubicin; metastatic breast cancer

Doxorubicin is among the most effective chemotherapeutic drugs in the treatment of metastatic breast cancer. Used as a single agent, doxorubicin induces response rates of approximately 40% as first-line chemotherapy and about 20% as second-line therapy after failure or relapse on combination chemotherapy (Tormey et al, 1975; Hoogstraten et al, 1976).

Although the acute toxicities of doxorubicin are manageable, the major cumulative dose-limiting toxic effect of the drug is the development of congestive heart failure (CHF), which may be irreversible and lethal (Lefrak et al, 1975; Von Hoff et al, 1979). The risk of CHF induction with doxorubicin is limited at a cumulative dose of less than 550 mg $m^{-2}$, but increases rapidly thereafter, preventing the continuation of the use of the drug beyond this dose. Since the early 1970s, there has been a continuous search for anthracycline analogues with a more favourable therapeutic profile than doxorubicin. A number of anthracycline derivatives of lower cardiotoxic potential in animal models have been introduced into clinical trials, of which one is epirubicin, an analogue resulting from the epimerization of 4′-hydroxyl of doxorubicin (Rozencweig et al, 1979). The mechanism of action of epirubicin is similar to that of doxorubicin: binding to DNA and inhibiting synthesis and function of nucleic acid (Young and Weenen, 1984).

Experimental and phase I studies in breast cancer suggested that epirubicin had a more favourable therapeutic index than doxorubicin, i.e. similar anti-tumour activity but less toxicity (Casazza et al, 1980; Schrauer et al, 1981; Bonfante et al, 1982; Young and Weenen, 1984). In the early phase II studies in metastatic breast cancer, epirubicin showed anti-tumour activity similar to that of doxorubicin (Rozencweig et al, 1984). The toxicities encountered with epirubicin therapy were: leucopenia, nausea and vomiting and no severe life-threatening cardiac toxicity up to a cumulative dose of about 1000 mg $m^{-2}$.

In view of these early data, the EORTC Breast Cancer Cooperative Group decided to conduct a randomized phase II/III study directly comparing epirubicin with doxorubicin. The objectives of the study were to assess the objective anti-tumour activity and the toxic effects of epirubicin, and to evaluate the cross-resistance between the anthracycline analogues. Both drugs were given as single agents in patients with metastatic breast cancer relapsing after previous chemotherapy without anthracyclines. The dose levels selected for epirubicin and doxorubicin were 90 mg $m^{-2}$ and 75 mg $m^{-2}$ respectively. Both drugs were administered as an i.v. bolus injection every 3 weeks. Based on the limited data, available at that time, of previous investigations, epirubicin 90 mg $m^{-2}$ was anticipated to induce degrees of myelosuppression equivalent to doxorubicin 75 mg $m^{-2}$ (Rozencweig et al, 1984; Bonfante et al, 1982). Haematological growth factors were not used in this study.

*Received 16 May 1997*
*Revised 8 December 1997*
*Accepted 9 December 1997*

*Correspondence to:* M Bontenbal, Department of Medical Oncology, Rotterdam Cancer Institute (Dr Daniel den Hoed Kliniek), PO Box 5201, 3000 AE Rotterdam, The Netherlands

### MATERIALS AND METHODS

Patients with metastatic breast cancer were eligible for this study, after giving informed consent according to the rules of the participating institution.

Measurable or evaluable progressive disease was required as well as a performance status (WHO) better than 3. Patients with liver enlargement, pleural effusion, ascites, bone marrow involvement or osteoblastic lesions were not considered to be evaluable. In addition, patients were excluded if they had received more than one previous combination chemotherapy regimen without anthracyclines, as adjuvant therapy or for metastatic disease. Other exclusion criteria included renal (creatinine > 1.2 mg $dl^{-1}$) and/or hepatic (bilirubin > 1.5 mg $dl^{-1}$) dysfunction, congestive heart failure, significant arrhythmia, bilateral bundle branch block or history of myocardial infarction as well as previous or concurrent malignancies (except adequately treated carcinoma in situ of the cervix and/or carcinoma of the skin).

### Study design

Eligible patients were randomized between epirubicin and doxorubicin treatment by telephoning the EORTC data centre. Patients were stratified by institution. Patients failing either epirubicin or doxorubicin, after two, three or four courses, were to be crossed over to doxorubicin or epirubicin respectively. Originally the study was started as a randomized phase II trial, but after an interim analysis had been performed, it was continued as a phase III trial.

### Treatment protocol

The doses of epirubicin and of doxorubicin were 90 mg $m^{-2}$ and 75 mg $m^{-2}$ respectively. Both drugs were administered as an i.v. bolus injection and cycles were repeated every 3 weeks.

### Dose modifications

Treatment was delayed by 1 week if WBC was < 3 × $10^9$ $l^{-1}$ or platelets < 100 × $10^9$ $l^{-1}$ at the scheduled time of the subsequent cycle. Further dose adjustments were made as follows: 50% of the dose if after a 1-week delay the WBC count was between 2 and 2.9 × $10^9$ $l^{-1}$ and/or platelets between 50 and 99 × $10^9$ $l^{-1}$; or postponement for another week if the WBC count was < 2 × $10^9$ $l^{-1}$ or platelets < 50 × $10^9$ $l^{-1}$. If postponement was required for more than 3 weeks the patient went off study. The dose was also reduced to 50% if bilirubin level ranged between 2 and 3 mg $dl^{-1}$ and to 0% with a bilirubin level above 3 mg $dl^{-1}$. Other reasons for withdrawal from treatment were patients refusal or persistent severe side-effects other than haematological toxicity, congestive heart failure and neutropenic sepsis.

### Treatment duration

Patients with remission or stable disease after two courses continued treatment until the disease progressed. On disease progression, patients on epirubicin or on doxorubicin having received less than five courses were crossed over to doxorubicin or epirubicin respectively. Treatment was stopped at a cumulative dose of 550 mg $m^{-2}$ for doxorubicin and, initially, also for epirubicin. After 100 patients had been entered, the maximum epirubicin cumulative dose was increased to 900 mg $m^{-2}$.

**Table 1** Patient characteristics at entry (all eligible patients)

| | Doxorubicin (*n* = 118) | Epirubicin (*n* = 114) |
|---|---|---|
| Median age (range) (years) | 56 (31–75) | 56 (34–73) |
| Performance status WHO (*n*) | | |
| 0 | 38 (32)[a] | 35 (31) |
| 1 | 49 (42) | 53 (46) |
| 2 | 28 (24) | 25 (22) |
| Unknown | 3 (2) | 1 (1) |
| Prior endocrine therapy | | |
| None | 31 (26) | 33 (29) |
| Ablative | 22 (19) | 19 (17) |
| Additive | 43 (36) | 48 (42) |
| Both | 13 (11) | 10 (9) |
| Unknown | 9 (8) | 4 (4) |
| Prior chemotherapy | 115 (98) | 110 (97) |
| Dominant site | | |
| Soft tissue | 37 (31) | 27 (24) |
| Bone | 32 (27) | 31 (27) |
| Visceral | 49 (42) | 56 (49) |
| Menopausal status | | |
| Before menopause | 0 (0) | 4 (4) |
| Natural menopause | 71 (60) | 80 (70) |
| Artificial menopause | 38 (32) | 25 (22) |
| Unknown | 9 (8) | 5 (4) |

[a]Numbers in parentheses are percentages.

**Table 2** Treatment duration and dosages (all eligible patients who started the treatment)

| | Doxorubicin (*n* = 116) | Epirubicin (*n* = 113) |
|---|---|---|
| Duration of treatment (days) | | |
| Median (range) | 127 (1–314) | 113 (1–323) |
| No. with information | 116 | 113 |
| Dosage | | |
| Total dose (mg) | | |
| Median (range) | 568 (80–1476) | 800 (140–2250) |
| No. with information | 101 | 97 |
| Total dose (mg $m^{-2}$) | | |
| Median (range) | 383 (47–911) | 447 (88–1452) |
| No. with information | 92 | 93 |
| Total dose (mg $m^{-2}$) $week^{-1}$) | | |
| Median (range) | 23 (10–38) | 27 (12–49) |
| No. with information | 90 | 92 |
| Total dose as a percentage of the planned dose | | |
| Median (range) | 90 (41–154) | 91 (40–162) |
| No. with information | 90 | 92 |
| Relative dose intensity (*n*) | | |
| < 70% | 14 (16)[a] | 13 (14) |
| 70–89% | 30 (33) | 31 (34) |
| 90–109% | 32 (36) | 35 (38) |
| ≥ 110% | 14 (16) | 13 (14) |
| No. with information | 90 | 92 |
| Reductions | | |
| No | 52 (58) | 64 (70) |
| At least once | 38 (42) | 28 (30) |
| Delay | | |
| No | 42 (47) | 51 (55) |
| At least once | 48 (53) | 41 (45) |

[a]Numbers in parentheses are percentages.

© Cancer Research Campaign 1998

**Table 3** Response rate (all eligible patients)

| | Doxorubicin (*n* = 118) | | Epirubicin (*n* = 114) | |
|---|---|---|---|---|
| | *n* | % | *n* | % |
| Complete response | 5 | 4 | 2 | 2 |
| Partial response | 38 | 32 | 30 | 26 |
| No change | 37 | 31 | 45 | 40 |
| Progression/early death/not evaluable | 38 | 32 | 37 | 33 |
| Progression | 18 | 15 | 23 | 20 |
| Early death due to malignant disease | 1 | 1 | 0 | 0 |
| Early death due to toxicity | 3 | 2 | 0 | 0 |
| Not evaluable | 16 | 14 | 14 | 13 |
| Two-sided *P*-value for trend | | | 0.324 | |
| Percentage of responders | | | | |
| CR or PR | 43 | 36 | 32 | 28 |
| No objective response | 75 | 64 | 82 | 72 |
| Two-sided *P*-value for comparison of the percentage of response | | | 0.173 | |

### Pretreatment and follow-up studies

Baseline investigations included history and physical examination, performance status, tumour measurements, complete blood count, chemistries, chest radiography, a bone scan or skeletal survey, electrocardiogram (ECG) and, preferentially, measurement of the isotopic left ventricular ejection fraction (LVEF). All baseline investigations were repeated after two courses and thereafter every 3–6 weeks. Chest radiography, bone scan and/or bone surveys were repeated every 12 weeks. WBC and platelet nadirs were measured weekly during the first two treatment cycles.

### Evaluation of response and toxicity

Patients were evaluable for response if they had received at least two courses of chemotherapy and if tumour measurements had been repeated at 6 weeks. Assessment of response was performed according to the UICC criteria (Hayward et al, 1977). Toxicity was assessed according to the WHO criteria (WHO, 1979). Duration of complete or partial response was measured from the date of randomization until the date of progressive disease. All cases were subjected to extramural review performed by both the study coordinator (AT van Oosterom) and an external reviewer with respect to eligibility and evaluability, treatment effectiveness, toxicity, the correct reporting of the data described in the files and their representation on the forms.

### Statistics

Assuming a response rate for doxorubicin of 30%, and to have a power of 0.85 to detect a difference of 20% for epirubicin, a sample size of 116 evaluable patients for each arm was required ($\alpha = 0.05$, $\beta = 0.15$, two-sided test).

The response to treatment and the degree of toxicity were compared using the chi-square test for proportions and the chi-square test for linear trend. Leucocyte and platelet values were compared using the Wilcoxon rank-sum test. Progression and survival curves were calculated based on the Kaplan–Meier product-limit estimate and compared using the log-rank test. Adjustment for imbalances in prognostic factors was carried out

**Figure 1** Duration of response (months) in 75 responding patients. EPI, epirubicin; Dox, doxorubicin; *n*, numbers of patients; O, observed number of relapses

**Figure 2** Time to progression (months) in 232 eligible patients. EPI, epirubicin; Dox, doxorubicin; *n*, numbers of patients; O, observed number of relapses

**Figure 3** Overall survival (months) in 232 eligible patients. EPI, epirubicin; Dox, doxorubicin; *n*, numbers of patients; O, observed number of relapses by means of retrospective stratification. Except for patients lost to follow-up, all patients were followed until death.

### RESULTS

### Patient characteristics

Within this study (EORTC 10811) 259 patients were randomized by ten institutions between June 1982 and May 1986 – 128 patients to doxorubicin and 131 to epirubicin. Twenty-seven patients (ten on doxorubicin, 17 on epirubicin) were ineligible. Reasons for ineligibility were: prior or concomittant treatment ($n$ = 4), poor performance status ($n$ = 7), insufficient WBC count ($n$ = 3), cardiovascular disease ($n$ = 4), non-measurable disease ($n$ = 4), brain metastases ($n$ = 2), insufficient data ($n$ = 2) and previous endometrial cancer ($n$ = 1).

The characteristics of the 232 eligible patients are given in Table 1. The two treatment groups were well balanced with respect to age, performance status and prior hormono- or chemotherapy. For the dominant site, a small imbalance was observed: visceral lesions were more frequent in the epirubicin arm and there were more soft tissue lesions in the doxorubicin arm. Previous adjuvant therapy was not recorded on the forms, but prior chemotherapy had mainly been applied for advanced disease.

### Treatment duration and dosages

Three eligible patients never started their treatment. One patient in the doxorubicin arm had an episode of infection soon after randomization, and another one refused to start the treatment. One patient in the epirubicin arm died suddenly before the treatment was started.

The median number of treatment courses was five in both arms: range one to 14 cycles in the doxorubicin arm and one to 15 cycles in the epirubicin arm. Treatment duration and total dosages administered are given in Table 2. The median duration of treatment was 127 days in the doxorubicin arm (range 1–314 days) and 113 days in the epirubicin arm (range 1–323 days). The median dose of drug received per m$^2$ was 383 mg in the doxorubicin arm (range 47–911 mg) and 447 mg in the epirubicin arm (range 88–1452 mg). The median dose intensity was 90% in the doxorubicin arm (range 41–154%) and 91% in the epirubicin arm (range 40–162%). Fifty-three per cent of the patients in the doxorubicin arm and 45% of the patients in the epirubicin arm had at least one cycle delayed. Dose reductions occurred in 42% and 30% of the patients in the doxorubicin and epirubicin arms respectively. Twenty-one patients on doxorubicin and 16 on epirubicin stopped the treatment prematurely because of toxicity or treatment refusal. Eleven patients on epirubicin stopped the treatment while still responding at a median cumulative dose of 544 mg m$^{-2}$ (range 495–1452 mg m$^{-2}$, i.e. ≥ six cycles).

### Treatment efficacy

Including the non-evaluable patients, the response rate (CR + PR) was 36% for the doxorubicin arm (95% CI 28–45%) and 28% for the epirubicin arm (95% CI 20–36%, Table 3). This difference of 8% in the response rate (95% CI –3% to +20%) is not significant using the chi-square test ($P$ = 0.173). Likewise, it is not significant using a test for linear trend ($P$ = 0.324 for CR vs PR vs NC vs other). When stratified by the dominant site of the disease, there was no significant difference in response between both drugs ($P$ = 0.436). The duration of response among the complete (CR) and partial (PR) responders is presented in Figure 1. The median duration of response, as measured from the date of randomization, was 40 weeks for doxorubicin and 32 weeks for epirubicin. Using the log-rank test, this difference approaches significance ($P$ = 0.059).

Time to progression for all eligible patients is presented in Figure 2. The median time to progression was 23 weeks for doxorubicin and 19 weeks for epirubicin. Using the log-rank test with or without adjustment for dominant site, this difference is not statistically significant (unstratified $P$ = 0.063, stratified $P$ = 0.085). Figure 3 shows the duration of overall survival for all eligible patients. The median survival for patients treated with doxorubicin was 47 weeks and for patients treated with epirubicin 44 weeks (unstratified $P$ = 0.196, stratified $P$ = 0.385). An analysis of time to progression and duration of survival in all randomized patients yielded similar results. A total of five patients initially treated with epirubicin were crossed over to doxorubicin treatment because of progressive disease. None of these patients responded. Among nine patients not responding to first-line doxorubicin, one PR (11%) was observed with the use of second-line epirubicin treatment.

**Table 4** Haematological toxicity (all eligible patients who started the treatment)

| | Doxorubicin ($n$ = 116) | Epirubicin ($n$ = 113) | $P$-value[a] |
|---|---|---|---|
| *Leukocytes ($10^9$ $l^{-1}$)* | | | |
| Nadir over the whole treatment period | | | |
| Median (range) | 2.5 (0.2–3.9) | 2.8 (0.2–4.0) | |
| No. with information | 60 | 56 | 0.150 |
| Worst retreatment value over the whole treatment | | | |
| Median (range) | 3.6 (2.0–10.3) | 3.9 (2.3–12.7) | |
| No. with information | 103 | 107 | 0.011 |
| Nadir over the first two cycles | | | |
| Median (range) | 2.5 (0.2–3.9) | 2.7 (0.2–4.0) | |
| No. with information | 42 | 38 | 0.429 |
| Worst retreatment value over the first two cycles | | | |
| Median (range) | 4.0 (2.2–10.5) | 4.3 (2.5–17.5) | |
| No. with information | 103 | 107 | 0.030 |
| *Platelets ($10^9$ $l^{-1}$)* | | | |
| Nadir over the whole treatment period | | | |
| Median (range) | 88 (8–206) | 143 (27–335) | |
| No. with information | 24 | 22 | 0.031 |
| Worst retreatment value over the whole treatment | | | |
| Median (range) | 244 (50–592) | 266 (105–552) | |
| No. with information | 102 | 107 | 0.077 |
| Nadir over the first two cycles | | | |
| Median (range) | 77 (8–301) | 127 (27–335) | |
| No. with information | 19 | 17 | 0.163 |
| Worst retreatment value over the first two cycles | | | |
| Median (range) | 290 (50–656) | 279 (107–692) | |
| No. with information | 102 | 107 | 0.778 |

[a]Wilcoxon rank-sum test.

© Cancer Research Campaign 1998

**Table 5** Non-haematological toxicity (worst grade reported during the treatment period for all eligible patients who started the treatment)

| | Grade | | | | | Total | *P*-value |
|---|---|---|---|---|---|---|---|
| | 0 | 1 | 2 | 3 | 4 | | |
| Oral | | | | | | | |
| Doxorubicin | 57 | 26 | 24 | 7 | 0 | 114 | |
| Epirubicin | 89 | 13 | 10 | 1 | 0 | 113 | <0.001 |
| Diarrhoea | | | | | | | |
| Doxorubicin | 88 | 11 | 12 | 2 | 1 | 114 | 0.005 |
| Epirubicin | 103 | 4 | 6 | 0 | 0 | 113 | |
| Haemorrhage | | | | | | | |
| Doxorubicin | 104 | 5 | 4 | 1 | 0 | 114 | |
| Epirubicin | 111 | 0 | 2 | 0 | 0 | 113 | 0.048 |
| Nausea/vomiting | | | | | | | |
| Doxorubicin | 9 | 20 | 46 | 31 | 8 | 114 | |
| Epirubicin | 6 | 28 | 49 | 26 | 4 | 113 | 0.304 |
| Fever | | | | | | | |
| Doxorubicin | 90 | 11 | 12 | 0 | 2 | 115 | |
| Epirubicin | 93 | 12 | 8 | 0 | 0 | 113 | 0.215 |
| Alopecia | | | | | | | |
| Doxorubicin | 10 | 4 | 15 | 81 | 2 | 112 | |
| Epirubicin | 15 | 2 | 13 | 80 | 1 | 111 | 0.483 |
| Infection | | | | | | | |
| Doxorubicin | 89 | 14 | 9 | 1 | 2 | 115 | |
| Epirubicin | 93 | 9 | 6 | 3 | 1 | 112 | 0.546 |

### Toxicity

The haematological toxicity data are presented in Table 4. They are expressed either as nadir or as retreatment day (first day of next cycle) values, calculated as the worst reported value over all cycles. The difference in the lowest leucocyte count on retreatment day was significant between the doxorubicin and the epirubicin arm ($P = 0.011$), with a lower value in the former arm. The platelet nadir (based on 46 patients) was significantly lower in the doxorubicin arm ($P = 0.031$). Three patients died in the doxorubicin arm with infectious complications during leucopenia, whereas no toxic deaths were observed in the epirubicin arm. The data for the non-haematological toxicities are presented in Table 5, displaying the maximal toxicity assessed per patient, excluding information after crossing over.

Mucositis and diarrhoea were significantly lower in the epirubicin-treated patients than in the doxorubicin-treated patients ($P < 0.001$ and $P = 0.005$ respectively). There were also fewer haemorrhages with epirubicin than with doxorubicin ($P = 0.048$).

Concerning cardiotoxicity, nine patients in the doxorubicin arm and two patients in the epirubicin arm experienced clinical congestive heart failure (CHF). However, sporadic measurements of LVEF precluded detailed assessment of cardiotoxicity.

### DISCUSSION

There is virtually no cure for disseminated breast cancer. Therefore, treatment of metastatic disease is palliative and aims at symptom relief and prolongation of life. In this situation, side-effects of therapy should be minimal.

The objective of the present study (started in the 1980s) was to compare the activity and toxicity of doxorubicin with its presumed equally effective but less toxic derivate epirubicin as second-line chemotherapy in patients with metastatic breast cancer. To the best of our knowledge, this is the largest randomized trial directly comparing the two anthracyclines in advanced breast cancer. Eight smaller randomized studies in metastatic breast cancer have compared the efficacy of monotherapy, with both drugs given on an equimolar or equimyelotoxic basis (Table 6). In the four studies comparing epirubicin with doxorubicin on an equimolar basis, no differences in response percentages between the two treatment arms were observed (Brambilla et al, 1986; Perevodchikova and Valvere, 1987; Lawton et al, 1990; Gasparini et al, 1991). However, the numbers of patients included in those studies were small, precluding any firm conclusions. Toxicity, however, was generally more pronounced in the doxorubicin-treated patients. The four other studies compared the two drugs on an expected equimyelotoxic basis with doses of epirubicin ranging from 1.4 to 1.5 times the dose of doxorubicin (Jain et al, 1985; Taguchi et al, 1986; Hortobagyi et al, 1989; Perez et al, 1991). Similarly none of these studies showed significant differences in response rates, duration of response or survival between the two treatment arms.

The efficacy of equimolar doses of epirubicin and doxorubicin as part of a drug combination with fluorouracil and cyclophosphamide (FEC vs FAC) was investigated in two large randomized trials (The French Epirubicin Study Group, 1988; The Italian Multicentre Breast Study with Epirubicin, 1988). Response percentages, duration of

**Table 6** Doxorubicin vs epirubicin as single-agent therapy in metastatic breast cancer

| Main author | Year | No. of patients | Dose (mg $m^{-2}$) | | Response rates (%) | |
|---|---|---|---|---|---|---|
| | | | Doxorubicin | Epirubicin | Doxorubicin | Epirubicin |
| Brambilla | 1986 | 42 | 75 | 75 | 52 | 62 |
| Perevodchikova | 1987 | 30 | 90 | 90 | 33 | 33 |
| Lawton | 1990 | 56 | 70 | 70 | 36 | 32 |
| Gasparini | 1991 | 43 | 20[a] | 20[a] | 38 | 36 |
| | | | | | | |
| Jain | 1985 | 52 | 60 | 85 | 25 | 25 |
| Taguchi | 1986 | 63 | 40 | 60 | 35 | 56 |
| Hortobagyi | 1989 | 48 | 60 | 90 | 29 | 26 |
| Perez | 1991 | 138 | 60 | 90 | 47 | 49 |
| Bontenbal | 1996 | 233 | 75 | 90 | 36 | 28 |

[a]Weekly administrations.

response and time to progression did not differ in the two treatment arms. However, the epirubicin combination showed a lesser degree of myelosuppression, nausea and vomiting.

In our study, no significant differences in response rates were observed between the two drugs, with 36% compared with 28% objective responses (CR + PR) for doxorubicin and epirubicin respectively. However, the study was designed to detect a difference in response in favour of epirubicin of 20% in 232 patients. Because the difference in response is smaller, a much larger study would be needed to detect a possible clinically important difference in response between both drugs. There was at best a trend in favour of doxorubicin for the median duration of response (40 vs 30 weeks) and for the median time to progression (23 vs 19 weeks), but the differences were small and duration of survival was the same in both groups. An explanation for the small differences we observed in time to progression and duration of response can possibly be found in the initial design of the study, in that treatment had to be stopped after a cumulative dose of 550 mg $m^{-2}$ was reached for both drugs. Indeed, 11 patients in the epirubicin arm who had experienced a response stopped treatment after a cumulative dose of 495–1452 (median 544) mg $m^{-2}$. Reduction of the treatment period could have influenced the time to progression and duration of response, as was found in the studies of Ejlertsen et al (1993) and Coates et al (1987). On the other hand, dose reductions occurred more frequently in patients treated with doxorubicin (42% vs 30%).

We observed a statistically significant difference in toxicity in favour of epirubicin. Doxorubicin treatment resulted in more bone marrow depression, mucositis and diarrhoea. In the study of Perez et al (1991), bone marrow toxicity of doxorubicin 60 mg $m^{-2}$ and epirubicin 90 mg $m^{-2}$ were almost superimposable, and the same results were found for gastrointestinal toxicity. Response percentages in this study were indentical, and the authors concluded that the higher dose of epirubicin had no advantages over the lower dose of doxorubicin. The increased bone marrow toxicity seen with doxorubicin gives rise to the question of whether we used an insufficient dose of epirubicin.

Bone marrow toxicity is historically used to relate the dose of one drug to another (Launchbury and Habboubi, 1993). At the time this study started, clinical experience suggested that doxorubicin 75 mg $m^{-2}$ and epirubicin 90 mg $m^{-2}$ would induce similar degrees of myelotoxicity (Rozencweig et al, 1984; Bonfante et al, 1982). In 1990, Mouridsen reviewed 10 years of clinical experience with epirubicin and calculated the equitoxic dose ratio for the haematological toxicity of doxorubicin and epirubicin to be 1:1.2 (Mouridsen et al, 1990). Drug dosages chosen in this study fulfilled this criterium. Furthermore, Bastholt et al (1996) compared the efficacy and toxicity of four different dose levels of epirubicin in patients with metastatic breast cancer. An increase in the dose from 90 mg $m^{-2}$ to 135 mg $m^{-2}$ resulted in increased toxicity but had no impact on the efficacy of the drug. However, the number of patients in this study was small, and clinically important differences between these two doses could not be excluded.

We conclude from this study that a trend that approaches a significant difference in efficacy is achieved with doxorubicin 75 mg $m^{-2}$ and epirubicin 90 mg $m^{-2}$ in the treatment of advanced breast cancer. The equitoxic dose ratio for haematological toxicity of doxorubicin and epirubicin is $>1:1.2$, whereas the ratio for cardiotoxicity remains uncertain. On the other hand, using these dose levels, the treatment with epirubicin is associated with significantly fewer side-effects.

### ACKNOWLEDGEMENTS

We would like to thank our colleagues Dr TAW Splinter, Dr LVA Beex and Dr CAM de Swart for participating in this study, and Mrs FJ Smits for typing the manuscript.

### REFERENCES

Bastholt L, Dalmark M, Gjedde SB, Pfeiffer P, Pedersen D, Sandberg E, Kjaer M, Mouridsen HT and Rose C (1996) Dose–response relationship of epirubicin in the treatment of postmenopausal patients with metastatic cancer: a randomized study of epirubicin at four different dose levels performed by the Danish Breast Cancer Cooperative Group. *J Clin Oncol* **14**: 1146–1155

Bonfante V, Villani F and Bonadonna G (1982) Toxic and therapeutic activity of 4′epi-doxorubicin. *Tumori* **68**: 105–111

Brambilla C, Rossi A, Bonfante V, Ferrari L, Villani F, Crippa F and Bonadonna G (1986) Phase II study of doxorubicin versus epirubicin in advanced breast cancer. *Cancer Treat Rep* **70**: 261–266

Casazza AM, Dimarco A, Bonadonna G, Bonfante V, Bertazzoli C, Bellini O, Pratezi G, Salla L and Ballerini L (1980) Effects of modifications in position 4 of the chromophore or in position 4 of the aminosugar, on the antitumor activity and toxicity of daurorubicin and doxorubicin. In *Anthracyclines: Current Status and Developments*, Crooke ST and Reich SD. (eds), pp. 403–430. Academic Press: New York

Coates A, Gebski V, Bishop JF, Jeal PN, Woods RL, Snyder R, Tatter-Sall MHN, Byrne M, Harvey V, Gill G, Simpson J, Drummond R, Browne J, van Cooten R and Forbes JF (1987) Improving the quality of life during chemotherapy for advanced breast cancer – a comparison of intermittent and continuous treatment strategies. *N Engl J Med* **317**: 1490–1495

Ejlertsen B, Pfeiffer P, Pedersen D, Mouridsen HT, Rose C, Over-Gaard M, Sandberg E and Kristensen B (1993) Decreased efficacy of cyclophosphamide, epirubicin and 5-fluorouracil in metastatic breast cancer when reducing treatment duration from 18 to 6 months. *Eur J Cancer* **29A**: 527–531

The French Epirubicin Study Group (1988) A prospective randomized phase III trial comparing combination chemotherapy with cyclophosphamide, fluorouracil and either doxorubicin or epirubicin. *J Clin Oncol* **6**: 679–688

Gasparini G, Dal Fior S, Panizzoni GA, Favretto S and Pozza F (1991) Weekly epirubicin versus doxorubicin as second-line therapy in advanced breast cancer. *Am J Clin Oncol* **14**: 38–44

Hayward JL, Carbone PP, Heuson JC Kumaoka S, Segaloff and Rubens SD (1977) Assessment of response to therapy in advanced breast cancer. *Cancer* **39**: 1289–1294

Von Hoff DD, Layard MW, Base P, Davis HL, Von Hoff AL, Rozencweig M and Muggia F (1979) Risk factors for doxorubicin induced congestive heart failure. *Ann Intern Med* **91**: 710–717

Hoogstraten B, George SC, Samal B, Rivkin SE, Costanzi JJ, Bonnet JD, Thigpen T and Braine H (1976) Combination chemotherapy and adriamycin in patients with advanced breast cancer. *Cancer* **38**: 13–20

Hortobagyi GN, Yap HY, Kau SW, Fraschini MD, Ewer MS, Chawla SP and Benjamin RS (1989) A comparative study of doxorubicin and epirubicin in patients with metastatic breast cancer. *Am J Clin Oncol* **12**: 57–62

The Italian Multicentre Breast Study with Epirubicin (1988) Phase III randomized study of fluorouracil, epirubicin and cyclophosphamide v fluorouracil, doxorubicin and cyclophosphamide in advanced breast cancer: an Italian multicentre trial. *J Clin Oncol* **6**: 976–982

Jain KK, Casper ES, Geller NL, Hakes TB, Kaufman RJ, Currie V, Schwartz W, Cassidy C, Petroni GR, Young CW and Wittes R (1985) A prospective randomized comparison of epirubicin and doxorubicin in patients with advanced breast cancer. *J Clin Oncol* **3**: 818–826

Launchbury AP and Habboubi N (1993) Epirubicin and doxorubicin: a comparison of their characteristics, therapeutic activity and toxicity. *Cancer Treat Rev* **19**: 197–228

Lawton PA, Ostrowski MJ, Young T and Spittle MF (1990) Efficacy and toxicity of single agent chemotherapy in advanced breast carcinoma. *Br J Cancer* **61**: 177

Lefrak EA, Pitha J, Rosenheim S, O'Bryan RM, Burgess MA and Gottlieb JA (1975) Adriamycin (NSC 123127) cardiomyopathy. *Cancer Chemother Rep* **6**: 203–208

Mouridsen HT (1990) New cytotoxic drugs in treatment of breast cancer. *Acta Oncol* **29**: 343–347

Perevodchikova NI and Valvere VJ (1987) Comparative evaluation of farmorubicin and adriamycin in breast cancer. In *Progress in Antimicrobial and Anticancer Chemotherapy*, Berkada B. (ed.), pp. 474–477. Ecomed: Istanbul

© Cancer Research Campaign 1998

Perez DJ, Harvey VJ, Robinson BA, Atkinson PJ, Dady PJ, Kirk AR, Evans BD and Chapman PJ (1991) A randomized comparison of single-agent doxorubicin and epirubicin as first-line cytotoxic therapy in advanced breast cancer. *J Clin Oncol* **9**: 2148–2152

Rozencweig M, De Sloover C, Von Hoff DD, Tagnon HJ and Muggia FM (1979) Anthracycline derivates in new drug development programs. *Cancer Treat Rep* **63**: 807–808

Rozencweig M, Ten Bokkel Huinink WW, Cavalli F, Beunch U, Dombernowsky P, Holst H, Bramwell V, Renard J, Van Glabbeke M, Deesster G and Clarisse A (1984) Randomized phase II trial of carcinomycin vs 4-cpi-adriamycin in advanced breast cancer. *J Clin Oncol* **2**: 275–281

Schrauer PK, Wittes RE, Gralla RJ, Casper ES and Young CW (1981) A phase I trial of 4′epi-adriamycin. *Cancer Clin Trials* **4**: 433–437

Taguchi T, Ogawa M, Izuo M, Terasawa T, Yoshida M and Nakajima M (1986) A prospective randomised trial comparing epirubicin and doxorubicin versus epirubicin and doxorubicin in advanced breast cancer. *Jpn J Cancer Chemother* **13**: 3498–3507

Tormey DC (1975) Adriamycin (NSC-123127) in breast cancer: an overview of studies. *Cancer Chemother Rep* **6**: 319–327

WHO (1979) WHO Handbook for Reporting Results of Cancer Treatment, World Health Organization Offset Publication no. 48. WHO: Geneva

Young CW and Weenen H (1984) Pharmacology of epirubicin. In *Advances in Anthracycline Chemotherapy: Epirubicin*, Bonadonna G. (ed.), pp. 51–63. Masson: Miland

© Cancer Research Campaign 1998